# The Potential Role of Haploblocks in the Persistence of Small, Isolated Populations of Brook Trout (*Salvelinus fontinalis*)

**DOI:** 10.1002/ece3.72075

**Published:** 2025-09-15

**Authors:** Cait M. Nemeczek, M. Lisette Delgado, Meg E. Smith, John MacMillan, Mallory Van Wyngaarden, Daniel E. Ruzzante

**Affiliations:** ^1^ Department of Biology Dalhousie University Halifax Nova Scotia Canada; ^2^ Inland Fisheries Division Nova Scotia Department of Fisheries and Aquaculture Halifax Nova Scotia Canada

**Keywords:** adaptation, chromosomal inversions, intraspecific variation, structural variants

## Abstract

Understanding the role of structural variants such as chromosomal inversions in local adaptation among small, isolated populations is an important addition to robust conservation strategies, as most studies investigating inversions to date have been conducted on high gene flow systems. Brook Trout ( *Salvelinus fontinalis* ), an economically important top sportfish, is extremely vulnerable to thermal stress. Local adaptation with respect to this trait warrants investigation as climate change accelerates the loss of cold‐stream ecosystems. We performed low‐coverage whole‐genome sequencing on 192 Brook Trout from 9 small, isolated streams in Nova Scotia, Canada, to assess genetic diversity within and among stream populations. We detected four structural variants in the three westernmost populations, which differ from all other streams in water temperature, streamflow, and surficial geology. The structural variants appear to be chromosomal inversions. These genomic regions exhibit high linkage disequilibrium, and PCA revealed the presence of three karyotypes. Redundancy analysis provides support for potential local adaptation, with temperature, pH, and streamflow being important predictors of genomic variance and statistically significant SNPs falling within potential inverted regions. Mitogenome analyses suggest that a single glacial lineage recolonized the region. Individuals carrying the potential chromosomal inversions exhibited one of five related mitochondrial DNA haplotypes, but these haplotypes were found also in individuals without the potential inversions, suggesting they arose post‐recolonization. The genetic differentiation among the nine surveyed Brook Trout populations persists even after exclusion of the potentially inverted regions, suggesting these regions do not control the population structure of these Brook Trout populations.

## Graphical abstract

Small brook trout populations inhabit streams of the North Mountain, Nova Scotia, where western streams have different environmental characteristics. Low‐coverage whole genome sequencing was performed on 192 brook trout, and analyses showed the presence of potential inversions among individuals of only western populations. These findings highlight the importance of intraspecific population variation and how it can contribute to population persistence.

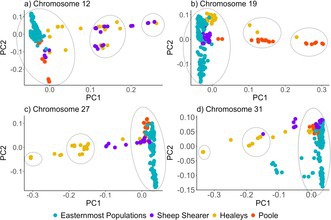

## 1 Introduction

Genetic diversity is an essential component of a species or population's ability to adapt to changing environmental conditions. Accordingly, it is included as a goal in the Convention on Biological Diversity (Convention on Biological Diversity 2022; Hoban et al. 2022) but is not yet widely implemented. Conservation management often focuses on the species level and can neglect the fine‐grained intraspecific variation found within and among populations. The IUCN Red List, for example, does not include intraspecific genetic diversity (Norderhaug et al. 2024) and, in addition, genetic monitoring of diversity can vary among regions (Pearman et al. 2024). However, intraspecific genetic diversity is used in conservation management with evolutionary significant units (Waples 1995) and designatable units (COSEWIC 2023). Local adaptation or phenotypic plasticity among populations, for example, can allow populations to persist in rapidly changing environments (Des Roches et al. 2018, 2021). Many recent studies highlight the importance of intraspecific variation (see Lujan et al. 2022; Shaney et al. 2020; von Takach et al. 2024) with some incorporating it into conservation management through conservation units and designatable units (see Lehnert et al. 2023; Waples et al. 2022). Genomic structural variants, including inversions, copy number variants, and transposable elements, are known to contribute significantly to the genetic variation observed within species (Dorant et al. 2020; Mérot et al. 2023; Wellenreuther et al. 2019; Wellenreuther and Bernatchez 2018) and are thus a critical component of intraspecific variation.

Chromosomal inversions are a type of structural variant that occur when part of the chromosome breaks, and the orientation of that segment becomes reversed (Kirkpatrick 2010). Research on chromosomal inversions has uncovered frequent and sometimes large chromosomal inversions with an average size of 8.4 megabases across species (An et al. 2024; Harringmeyer and Hoekstra 2022; Wellenreuther and Bernatchez 2018). When an inversion develops, genetic drift, mutations, and recombination will have varying effects that can cause changes in the inversion arrangement frequencies, leading to the maintenance, fixation, or removal of inversions over time (Faria, Johannesson, et al. 2019). Inversions can play a large role in local adaptation and speciation, which comes primarily from the suppressed recombination that takes place in heterokaryotypes carrying one copy of the inverted and one of the non‐inverted sequence arrangements. In some cases, inversions can reach fixation by selection or drift, leading to speciation (e.g., via selection against heterokaryotypes due to epistatic interactions). Chromosomal inversions can also be maintained by balancing selection, leading to polymorphism among populations (Faria, Johannesson, et al. 2019; Hoffmann and Rieseberg 2008; Kirkpatrick 2010). Recent research on seahorses ( *Hippocampus guttulatus* ) (Meyer et al. 2024) and stick insects (*Timema*) (Nosil et al. 2023) highlights situations in which inversions evolve and are maintained by factors including balancing selection. The role of inversions in diversification and local adaptation has also been well documented in monkey flowers ( *Mimulus guttatus* ) where they are responsible for annual and perennial ecotypes (Twyford and Friedman 2015); common quails ( *Coturnix coturnix* ) where a large inversion controls phenotypic differences in size, throat coloration, wings, migration, and flight efficiency (Sanchez‐Donoso et al. 2022). Chromosomal inversions have also been described in Atlantic Cod ( *Gadus morhua* ) where they appear to be linked to migratory behavior (Berg et al. 2016; Matschiner et al. 2022; Pampoulie et al. 2023; Sinclair‐Waters et al. 2018) as well as in willow warbler ( *Phylloscopus trochilus* ; Lundberg et al. 2023), Atlantic Herring ( *Clupea harengus* ; Fuentes‐Pardo et al. 2024; Han et al. 2020) and Atlantic Silversides ( *Menidia menidia* ; Akopyan et al. 2022), where they have been suggested to play a role in adaptation to environmental differences.

Inversions can be detected using a direct structural variant detection approach (Mérot et al. 2020) or an indirect detection approach (Faria, Chaube, et al. 2019; Mérot et al. 2021). The indirect approach involves the examination of patterns of linkage disequilibrium by chromosome, PCA, estimation of genetic differentiation with *F*_ST_, and an examination of patterns of heterozygosity within the groups identified in the PCA. As limited recombination can be indicative of a potential inversion, linkage disequilibrium patterns are the first step of this method. A PCA plot across individuals is expected to show three distinct genotype groups; the middle group comprising the heterokaryotypes, while the other two groups represent each homokaryotype as seen in recent studies (Huang et al. 2020; Nosil et al. 2023; Reeve et al. 2023). Heterozygosity is also used as supporting evidence for potential inversions, as heterozygous individuals will have one copy of the inverted arrangement and thus have higher heterozygosity than homokaryotypes.

Inversions have been discovered in several salmonid species including Atlantic salmon ( *Salmo salar* ) (Stenløkk et al. 2022), Lake Trout ( *Salvelinus namaycush* ) (Smith et al. 2020), Arctic Charr ( *Salvelinus alpinus* ) (Hale et al. 2021), Rainbow Trout ( *Oncorhynchus mykiss* ) (Pearse et al. 2019), and Chum Salmon ( *Oncorhynchus keta* ) (McKinney et al. 2020). However, studies of local adaptation and chromosomal inversions in Brook Trout are relatively scarce (Brookes et al. 2022; Jeon et al. 2025; Sutherland et al. 2016). Brook Trout, an economically important sportfish, are vulnerable to habitat loss, overexploitation, and invasive species as well as to climate change leading to decreased streamflow and concomitant increases in water temperature. This is a particularly challenging combination given the species' narrow thermal tolerance (Cherry et al. 1977; MacMillan et al. 2008; Nova Scotia Department of Agriculture and Fisheries Inland Fisheries Division 2005).

**FIGURE 1:**
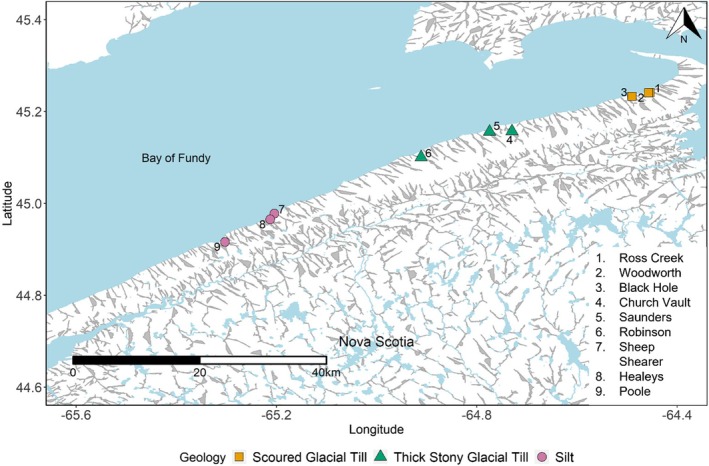
Nine streams in the North Mountain, Nova Scotia sampled for Brook Trout. Shapes indicate the type of surficial geology that makes up the streams.

Our primary objective in the present study was to examine the extent to which environmental variables influence genomic differentiation in Brook Trout. We examined genomic differentiation among nine pristine and isolated Brook Trout populations in the North Mountain region of Nova Scotia (Figure 1). These populations are largely isolated, with negligible to no gene flow among them (Ruzzante et al. 2016) with no documented evidence of having ever been stocked. Using low‐coverage whole‐genome sequencing (lcWGS) we found evidence of structural variants in a subset of the populations. Analysis of whole mitogenome data provided evidence suggesting the structural variants can be explained by post‐glacial dispersion history. We also explore genes within potential inversion regions and suggest possible roles in local adaptation. Knowledge of potential inversions and their role in local adaptation is expected to improve our understanding of Brook Trout population decline and help design a basis for new conservation efforts that take into consideration the potential existence of structural variants in isolated populations.

## 2 Materials and Methods

### 2.1 Sampling and Environmental Data

Brook Trout fin clip samples were collected from nine streams along the North Mountain (Nova Scotia) in June 2021 (*N* = 40 individuals per stream, total *N* = 360) and stored in 95% ethanol (Figure 1). These streams differ in environmental and physical characteristics such as surficial geology and pH (Tables S1 and S2). HOBO pendant temperature loggers were installed and kept (April–November 2021) on streambeds of five of the nine streams recording temperature every 2 h and maximum daily water temperatures calculated (Figure S1). Streamflow was measured using the float method in all nine streams once a month from July to November 2021 with an A‐Just‐A‐Bubble fishing float (Rainbow Plastics). pH was measured at the same time with a pHep^+^ H198108 (Hanna instruments).

### 2.2 DNA Extraction, Low‐Coverage Whole‐Genome Sequencing and Quality Control

DNA was extracted from *N* = 21–22 individuals per stream using the Omega E.Z.N.A tissue DNA kit (Omega Bio‐Tek) following the manufacturer's protocol. DNA was quantified using the Quanti‐iT Picogreen dsDNA assay (Invitrogen). Six libraries were prepared for low‐coverage whole‐genome sequencing with a target depth of 3× following the Illumina DNA Prep protocol with modifications of the Illumina Nextera‐Flex protocol, Hackflex (Gaio et al. 2022), multiplexed library prep from Baym et al. (2015), and the protocol from Therkildsen and Palumbi (2017). The six pooled libraries of 192 individuals were sequenced on four lanes of paired‐end 2 × 150 bp reads on a NovaSeq S4 flowcell (Illumina) at The Centre for Applied Genomics (The Hospital for Sick Children, Toronto, Ontario). Raw fastq files were assessed using FastQC v.0.11.9 (Andrews 2010) and MultiQC v.1.11 (Ewels et al. 2016) and i5 and i7 IDT Illumina and Illumina Nextera adapters were removed with Trimmomatic v.0.39 (Bolger et al. 2014) in paired‐end mode. Fastp v.0.23.1 (Chen et al. 2018) was used to remove poly‐G tails.

### 2.3 Genome Mapping and Quality Filtering

Filtered reads were mapped to the Brook Trout ( *Salvelinus fontinalis* ) chromosome level genome ASM2944872v1 (GenBank assembly accession: GCA_029448725.1) with a size of 2.5 Gbp and a complete reference mitogenome (NCBI Reference Sequence: NC_000860.1) size 16,624 bp with Bowtie2 v.2.4.4 (Langmead and Salzberg 2012). Unplaced scaffolds were not used for alignment to reduce the effects of reads mapping to duplicate locations. The indices of the reference genome and dictionary were generated using SAMtools v.1.13 (Li et al. 2009) and Picard tools v.2.26.3 (Picard Toolkit 2019). Aligned BAM files were filtered with SAMtools v.1.13 (Li et al. 2009) to remove discordantly mapped pairs and those with mapping quality scores below 20 before being merged. Picard tools v.2.26.3 (Picard Toolkit 2019) was used to identify and remove PCR and optical duplicate reads, and overlapping paired reads were removed using Bamutils v.1.0.14 (Jun et al. 2015). Filtered reads were aligned around indels with GATKs v.3.7 RealignerTargetCreator and IndelRealigner function (McKenna et al. 2010). Pilon v.1.24 (Walker et al. 2014) was used to detect and correct residual errors in the aligned mitogenomic sequences. Average read depth was calculated by stream (population) using SAMtools v.1.17 (Li et al. 2009) removing reads with a minimum base quality of < 20 and minimum mapping quality < 20. Average depth per individual for low‐coverage whole‐genome sequencing data was 2.0×–6.9×. Average depth per individual for mitogenomic samples was 391.4×–2484×.

### 2.4 Genotype Likelihoods and SNP Calling

Low‐coverage whole genome sequencing (lcWGS) generates only a few reads for each individual at a given location in the genome and, as such, ANGSD was used to calculate genotype likelihoods and allele frequencies. Scripts used are available on GitHub (https://github.com/cnemeczek). ANGSD v.0.940 (Korneliussen et al. 2014) ‐doDepth was used to get depth counts at every position for all populations together and populations individually and plotted as histograms in R v.4.3.0 (R Studio Team 2021) to select appropriate maximum and minimum depths for further ANGSD analyses. Briefly, the distribution of depth counts from the .depthGlobal files was plotted as histograms using R v.4.1.2 (R Studio Team 2021) with tidyr v.1.2.0 (Wickham and Girlich 2022), data.table v.1.14.2 (Dowle and Srinivasan 2021), ggplot2 v.3.3.6 (Wickham 2016), na.tools v.0.3.1 (Brown 2018), and dplyr v.1.0.9 (Wickham et al. 2022). Data was filtered based on the normal distribution, and the mean and standard deviation were calculated. The maximum and minimum depth were calculated based on the standard deviation of the mean. These parameters were used to estimate genotype likelihoods and allele frequencies. Global genotype likelihoods were calculated using the following parameters ‐uniqueOnly 1, ‐remove_bads 1, ‐only_proper_pairs 1, ‐trim 0, ‐C 50, ‐baq 1, ‐minInd 96, ‐minMapQ 20, ‐minQ 20, ‐setMinDepth 474, ‐setMaxDepth 676, ‐doCounts 1, ‐GL 1, ‐doGLF 2, ‐doMajorMinor 1, ‐doIBS 1, ‐doCov 1, ‐makeMatrix 1, ‐doMaf 1, ‐SNP_pval 1e‐6, ‐minMaf 0.05. SAMtools identified 2,620,282 SNPs from individuals aligned to the Brook Trout genome. The covariance matrices produced by ANGSD were used to perform a PCA in R v.4.1.2 (R Studio Team 2021) with ggplot2 v.3.3.5 (Wickham 2016), dplyr v.1.0.7 (Wickham et al. 2022), and tidyverse v.1.3.1 (Wickham et al. 2022) to examine evidence of population structure. Eigenvectors and values were calculated from the covariance matrices and used to plot principal components explaining the highest percentage of variation in the SNP data. A list of SNPs was extracted from the allele frequency .mafs.gz file produced by the global run of ANGSD. ANGSD was run again using the supplied SNP list to calculate genotype likelihoods and allele frequencies at those specific sites, and these were used for the estimation of linkage disequilibrium.

### 2.5 Genetic Differentiation

Genetic variation was assessed using *F*_ST_ estimated between populations with potential inversions versus those from populations without inversions to scan for outlier regions in the genome. Briefly, ANGSD v0.940 was used with ‐doSaf to produce unfolded sample allele frequencies. ANGSD's realSFS was used with ‐fold 1 to generate the folded site frequency spectrum between populations with and without potential inversions. ANGSD's realSFS fst stats and stats2 function were used to calculate average *F*_ST_ in windows of 10,000 bp with a 10,000 bp slide, and a Manhattan plot was generated in R v4.3.3 (R Studio Team 2021) (Figure S2).

### 2.6 Linkage Disequilibrium

Regions of high linkage can be evidence of genomic structural variants, including inversions. Linkage disequilibrium was calculated with ngsLD v.1.1.1 (Fox et al. 2019) using a ‐max_kb_dist of 100 and the decay rate determined as 15 kb. Linkage disequilibrium was calculated again using ‐max_kb_dist of 15 kb, and SNPs in linkage disequilibrium were pruned using ngsLD's prune_graph.pl, resulting in 391,154 SNPs. ANGSD v0.940 was run with the pruned SNP list to calculate allele frequencies, and the resulting beagle file was subset to remove potential inversion chromosomes, resulting in 352,578 SNPs. PCAngsd v1.36.1 (Meisner and Albrechtsen 2018) was used to generate a covariance matrix from the beagle to assess population structure with PCA (Figure S3). For pruning, the minimum weight for assuming SNPs were connected based on *r* ^2^ values (‐‐min_weight) was set to 0.4 based on the linkage disequilibrium decay plot and was done by chromosome.

The existence of potential inversions was explored on a chromosome‐by‐chromosome basis following Mérot et al. (2020). ngsLD v.1.1.1 (Fox et al. 2019) was used to calculate linkage disequilibrium for each chromosome using ‐max_kb_dist 0 and ‐max_snp_dist of 0 while randomly sampling 50% of SNPs. The *r* ^2^ values from the linkage disequilibrium data were divided into percentiles and plotted as heatmaps in R v.4.0.2 (R Studio Team 2021) using ggplot2 v.3.3.6 (Wickham 2016) following scripts from Mérot et al. (2021). Heatmaps of each chromosome were used to find areas of high linkage disequilibrium (*r* ^2^ above ~0.6). A subset of each of chromosomes 12, 19, 27, and 31 (CM055694.1, CM055701.1, CM055709.1, CM055713.1) from the Brook Trout genome with blocks of linkage disequilibrium (*r* ^2^ above ~0.6) was further analyzed using PCA to assess for evidence of potential inversions in all individuals (3 groups representing homokaryotypes for the potential inversion, homokaryotypes without the potential inversion, and heterokaryotypes with one copy of the potential inversion). SNPs within linkage disequilibrium blocks were used to estimate genotype likelihoods using ANGSD v.0.940. PCA was performed in R Studio v.4.0.2 with the eigen function and plotted with ggplot2 v.3.3.6. Heatmaps were plotted for chromosomes 12, 19, 27, and 31 using all populations, and all populations excluding the westernmost streams (Poole, Healeys, Sheep Shearer).

### 2.7 Heterozygosity

Heterozygosity was assessed to further support evidence of potential inversions as individuals with potential inversions should exhibit relatively low heterozygosity. ANGSD v.0.940 was used to calculate allele frequencies and Hardy–Weinberg equilibrium with ‐doHWE on individuals representing the three potential inversion groups for each of Brook Trout chromosomes 12, 19, 27, and 31 using SNP lists for entire chromosomes. Observed heterozygosity (*H*_obs_) was calculated followed by average *H*_obs_ in 10 kb windows with a 10 kb slide using the R package WindowScanR (Tavares 2022) across entire chromosomes following scripts from (Mérot et al. 2021). *H*_obs_ was also calculated within potential inverted regions, and the average *H*_obs_ was calculated in windows the size of potential inversion regions. Boxplots were generated using R v.4.0.2 (R Studio Team 2021) and ggplot2 v.3.3.6 (Wickham 2016). The size of potential inversions could be visually inferred from plots of raw *H*_obs_ due to reduced levels of heterozygosity among potential inversion homokaryotypes. The SNP lists were adjusted accordingly from those based on linkage disequilibrium to encompass these regions for PCA of genotype likelihoods and heterozygosity. Patterns of relatively low observed heterozygosity were more variable for chromosomes 19, 27, and 31, and as such, we refined the region to coincide with the area of highest linkage disequilibrium (Figure 3). Heterozygosity was also calculated across the genome using all 2.6 million SNPs for 21 individuals from Poole, Healeys, and Sheep Shearer that represent the homokaryotypes with potential inversions to assess background levels of heterozygosity (Figure S4).

### 2.8 Mitochondrial DNA Haplotypes and Phylogeny

Analysis of mitochondrial DNA was conducted to assess the number of glacial lineages present and estimate haplotype diversity among all populations. Mitogenomic sequences from Pilon (Walker et al. 2014) were loaded into Geneious Prime 2023.2.1 (https://www.geneious.com) and aligned using the MUSCLE alignment tool. Thirty haplotypes were identified and extracted using the “Find Duplicates” function and named by frequency. A neighbor‐joining tree was constructed using Geneious Prime 2023.2.1 with default parameters, and bootstraps were calculated with 100,000 replicates. The Lake Trout ( *Salvelinus namaycush* ) reference complete mitogenome (NCBI Reference Sequence: NC_036392.1, 16,653 bp) was used as the outgroup. A median‐joining haplotype network was constructed using PopArt v.1.7 (Leigh and Bryant 2015) run with an epsilon value of 0.

### 2.9 Gene Annotation

Gene ontology analysis was conducted to identify gene functions. The UCSC genome browser (Kent et al. 2002) was used to identify genes within the potential inverted regions on chromosomes 12, 19, 27, and 31 of the Brook Trout genome. The resulting gene table was sorted in R v.4.3.3 (R Studio Team 2021) to search for unique gene symbols. The Brook Trout genome is not fully annotated and, as such, resulting loci not corresponding to any genes were removed from further analyses. Lists of gene symbols were searched in Metascape v.3.5 (Zhou et al. 2019) with input species as “any” and analysis as “human” to find GO terms of biological processes, pathways, and protein interactions.

### 2.10 Environmental Association Analysis

Redundancy analysis (RDA) was completed using the R package vegan (Oksanen et al. 2024) to assess the role of environmental variables on the genomic patterns of the four potential inversion chromosomes. Genotype likelihoods were converted to genotype calls using ‐doPlink in ANGSD v0.940 with ‐minMaf 0.10, ‐minInd 0.90, ‐setMinDetpth 2, and ‐setMaxDepth 5. This was done for each of the four potential inversion chromosomes. The resulting .tped files were converted to .raw format using plink v.2.0 (Purcell et al. 2007) with ‐recode A. Each raw genotype file had approximately 9% missing data, which was filled in with the most common genotype at each SNP. Water temperature data were available from May to November 2021 for five of the nine streams. The association analyses were thus conducted on these five streams with average water temperature, average pH, and average streamflow. All environmental variables were standardized to zero mean and unit variance with vegan in R (Oksanen et al. 2024). Temperature, streamflow, and pH were checked for correlations to ensure all variables had Pearson correlation coefficients < 0.70. SNPs that were three standard deviations away from the mean based on the loadings of the four RDAs were extracted as outliers to assess if any were located within potential inversion regions.

## 3 Results

### 3.1 Genome Mapping ANGSD SNP Calling/Genotype Likelihoods

**FIGURE 2:**
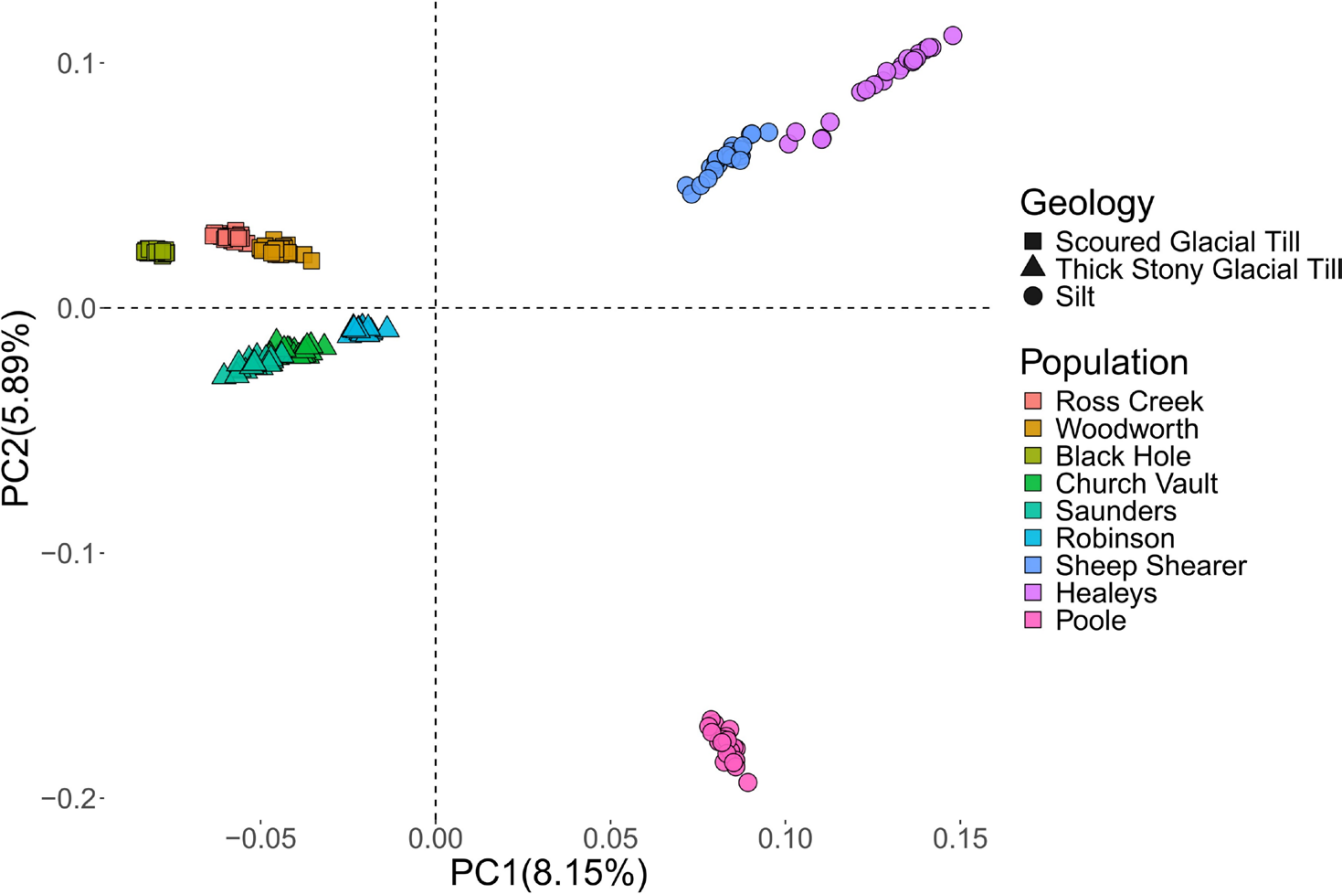
Principal component analysis plot using genotype likelihoods for *N* = 192 Brook Trout. Covariance matrix based on 2,620,282 SNPs found with reads aligned to the Brook Trout genome.

Target sequencing depth per individual was 3× and the average read depth per stream (population) after filtering out low‐quality reads was 2.2×–3× (Figure S5). PCA representing genotype likelihoods from more than 2.6 million SNPs shows populations separating along the first and second axes, with PC1 representing 8.15% of total variance and PC2 5.89% (Figure 2). This corresponds to differences in surficial geology where populations in silt habitats differ from those in scoured and thick stony glacial tills along PC1, and those in the other two types of glacial till (scoured vs. thick stony) differ from each other along PC2. Within the group of populations in the silt substrate, Poole differs from Sheep Shearer and Healey's along PC2 (Figure 2).

### 3.2 Potential Inversion Discovery and Support

#### 3.2.1 Linkage Disequilibrium

**FIGURE 3:**
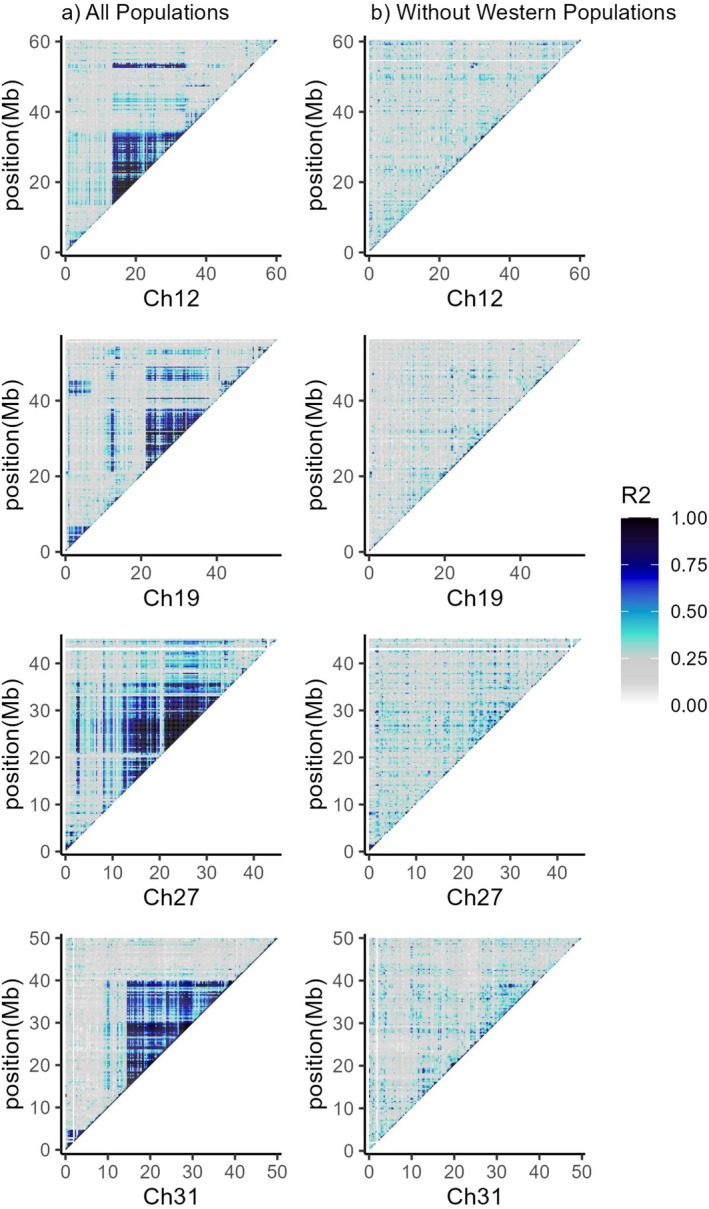
Heatmaps of chromosomes 12, 19, 27, and 31 (CM055694.1, CM055701.1, CM055709.1, and CM055713.1) from the Brook Trout genome. *r* ^2^ values in 250 kb windows of the second percentile from LD calculations are shown. (a) Heatmaps of all nine populations pooled (b) six populations excluding individuals from western streams (Healeys, Sheep Shearer, Poole). High *r* ^2^ values are those with dark colors and represent areas of high linkage disequilibrium. Regions of high linkage disequilibrium disappear when the three westernmost brook trout populations are excluded as shown in (b).

Heatmaps of LD for all populations pooled show multiple blocks of LD (*r* ^2^ > 0.60) with distinct blocks seen on each of chromosomes 12, 19, 27, and 31 (CM055694.1, CM055701.1, CM055709.1, CM055713.1) (Figure S6). All four LD blocks are larger than 10 Mb (Table 1). The patterns of large LD blocks disappear when Brook Trout from the three westernmost populations (Healeys, Sheep Shearer, Poole brooks) are excluded from the analysis (Figure 3) suggesting the large LD blocks are present exclusively in the three westernmost populations.

**TABLE 1:** Size of linkage disequilibrium blocks on chromosomes from the Brook Trout genome.

Chromosome	Region of LD block (mb)	Size of potential inversion (mb)
12 (CM055694.1)	14–35	18
19 (CM055701.1)	22–39	12
27 (CM055709.1)	13–35	12
31 (CM055713.1)	15–40	26

*Note:* The size was estimated from the *r* ^2^ of linkage disequilibrium shown in heatmaps for these chromosomes using all 192 Brook Trout. Size of potential inversions based on raw heterozygosity data.

#### 3.2.2 Genotype Likelihoods for Potential Inversion Regions

**FIGURE 4:**
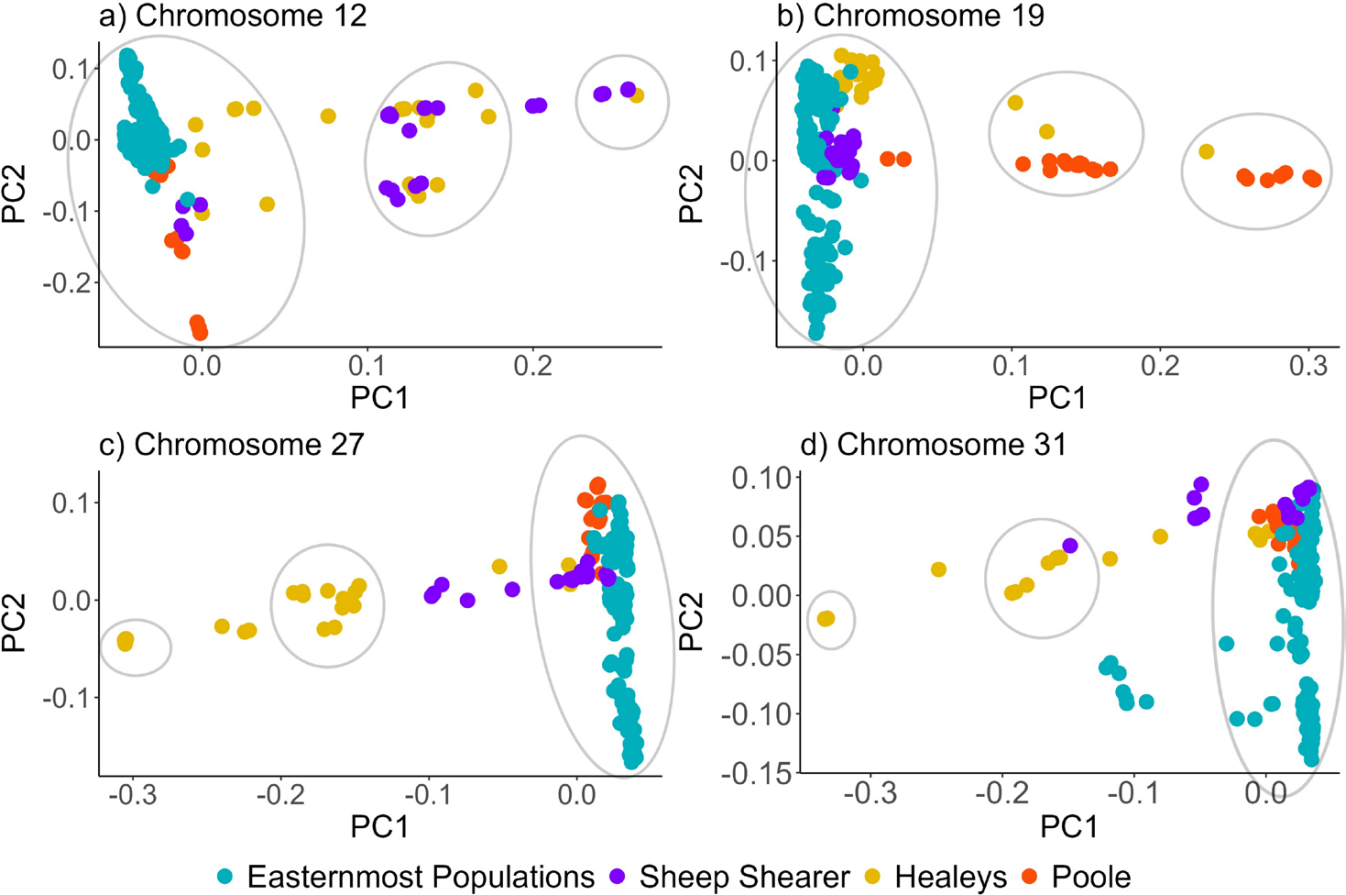
(a–d) PC plots for linkage disequilibrium blocks on Chromosomes 12, 19, 27, and 31 of the Brook Trout genome. The three clusters in (a–d) represent the different karyotypes expected when there is an inversion. The middle group on each panel represents the heterokaryotypes which have one copy of the potential inversion and one of the potential non‐inverted chromosome; the left and right groups represent either individuals with two copies of the potential inversion arrangement or no potential inversion arrangements. Percent variation explained for chromosome 12 PC1 19.6%, PC2 6.0%, chromosome 19 PC1 18.5%, PC2 6.5%, chromosome 27 PC1 20.9%, PC2 6.2%, chromosome 31 PC1 17.3%, PC2 6.4%. Only the three westernmost Brook Trout populations have individuals in the three karyotypes.

PCA plots of chromosomes 12, 19, 27, and 31 involving all nine populations are consistent with the results above (Figure 4). While three genotype groups are shown, with individuals clustering as homokaryotypes on the left and right sides of each panel, and heterokaryotypes in the middle (Figure 4) only the individuals from the three western populations (Sheep Shearer, Healeys, Poole) are represented in each of the three clusters: homokaryotypes with and without potential inversions and heterokaryotypes (Figure 4). In all four chromosomes, one homokaryotype group has ≤ 8 individuals. On chromosome 12, Brook Trout are found in each of the three genotype groups in Sheep Shearer and Healeys but not in Poole (Figure 4a). This indicates that both Sheep Shearer and Healeys have Brook Trout with both homokaryotypes and heterokaryotypes. A similar pattern is seen on chromosome 19 (Figure 4b). Brook Trout are found in each of the three genotype groups in Poole and Healeys but not in Sheep Shearer (Figure 4b). On chromosomes 27 and 31, only Brook Trout from Healeys brook are found in each of the three genotype groups, suggesting individuals in this brook are homozygous for the potential inverted and non‐inverted arrangements and heterozygous for potential inversion (Figure 4c,d).

#### 3.2.3 Heterozygosity

**FIGURE 5:**
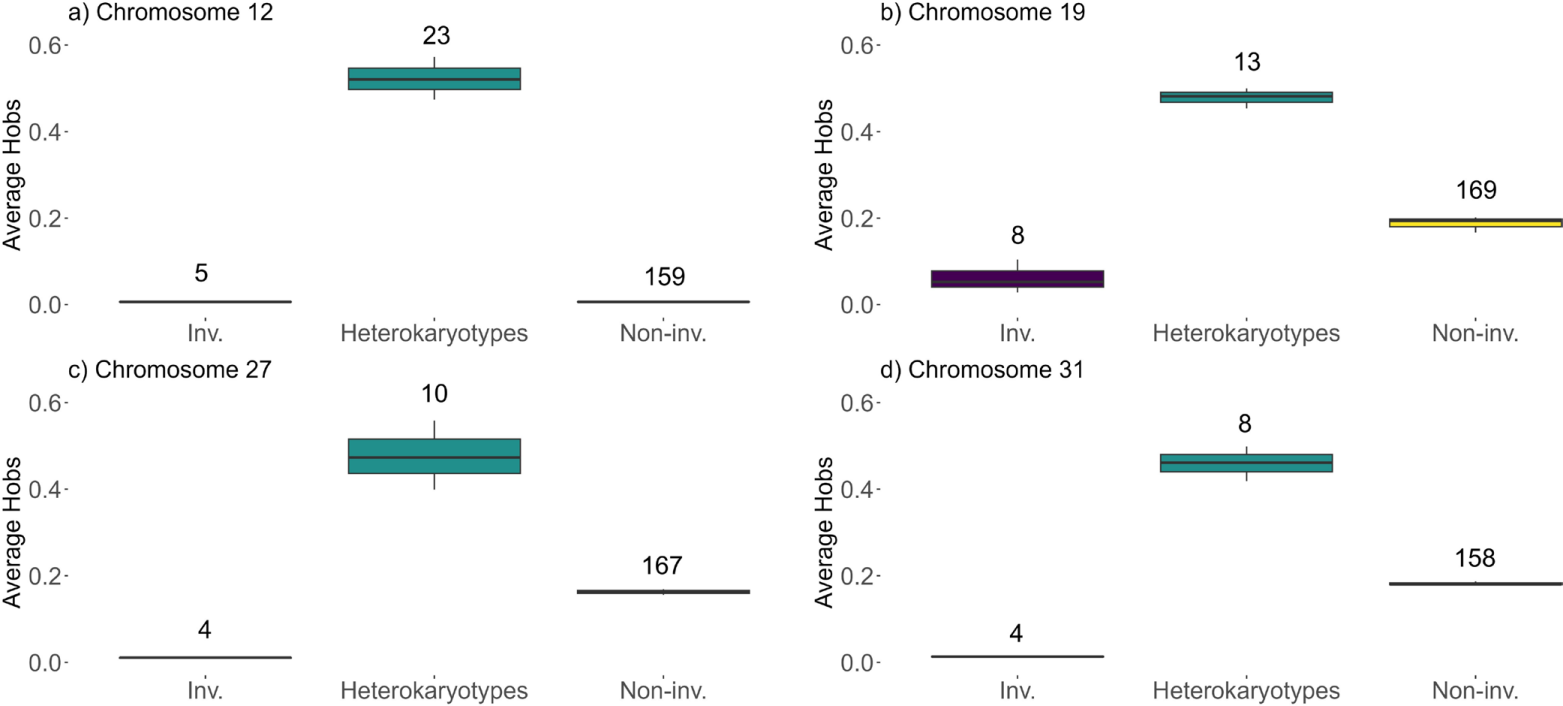
Average observed proportion of heterozygotes (*H*_obs_) in windows the size of potential inversion regions in (a) Chromosome 12, (b) Chromosome 19, (c) Chromosome 27, and (d) Chromosome 31. Individuals included in each of homokaryotypes with potential inversions (Inv.), heterokaryotypes, and homokaryotypes without potential inversions (Non‐inv.) are those represented by the three different karyotype groups in PCAs of linkage disequilibrium blocks (Figure 4). Number of individuals in each group is indicated above each bar. *H*_obs_ was estimated at each SNP and then averaged over the entire inversion region. Heterozygosity was consistently lowest among the homokaryotypes in the left group, which were presumed to be the inverted homokaryotypes.

The potential inversion breakpoints were estimated from *H*_obs_ plots across entire chromosomes due to the observable lower heterozygosity among potential inversion homokaryotypes (Figure S7). The sizes of the potential inversions are between 12 and 26 mbp (Table 1). Average *H*_obs_ within potential inversion regions on each of the four chromosomes is highest among heterozygous individuals for the inversion as these individuals have one copy of the non‐inverted arrangement and one copy of the potentially inverted arrangement, intermediate among individuals without the potential inversions with two copies of the non‐inverted arrangement and lowest among individuals with two copies of the potential inversion arrangement (Figure 5).

### 3.3 Genetic Differentiation and Structure

A Manhattan plot of *F*_ST_ between individuals from populations with potential inversions (Poole, Healeys, Sheep Shearer) and the six populations without potential inversions at all 2.6 million SNPs shows genome‐wide patterns of high *F*_ST_ with no distinct peaks that could represent regions under selection (Figure S2). A PCA of SNPs not in linkage and not from chromosomes with potential inversions shows a very similar pattern as that with all 2.6 million SNPs, suggesting genome‐wide differences that are not the result of potential inversions or other regions that could be under selection (Figure S3).

### 3.4 Haplotype Diversity

**FIGURE 6:**
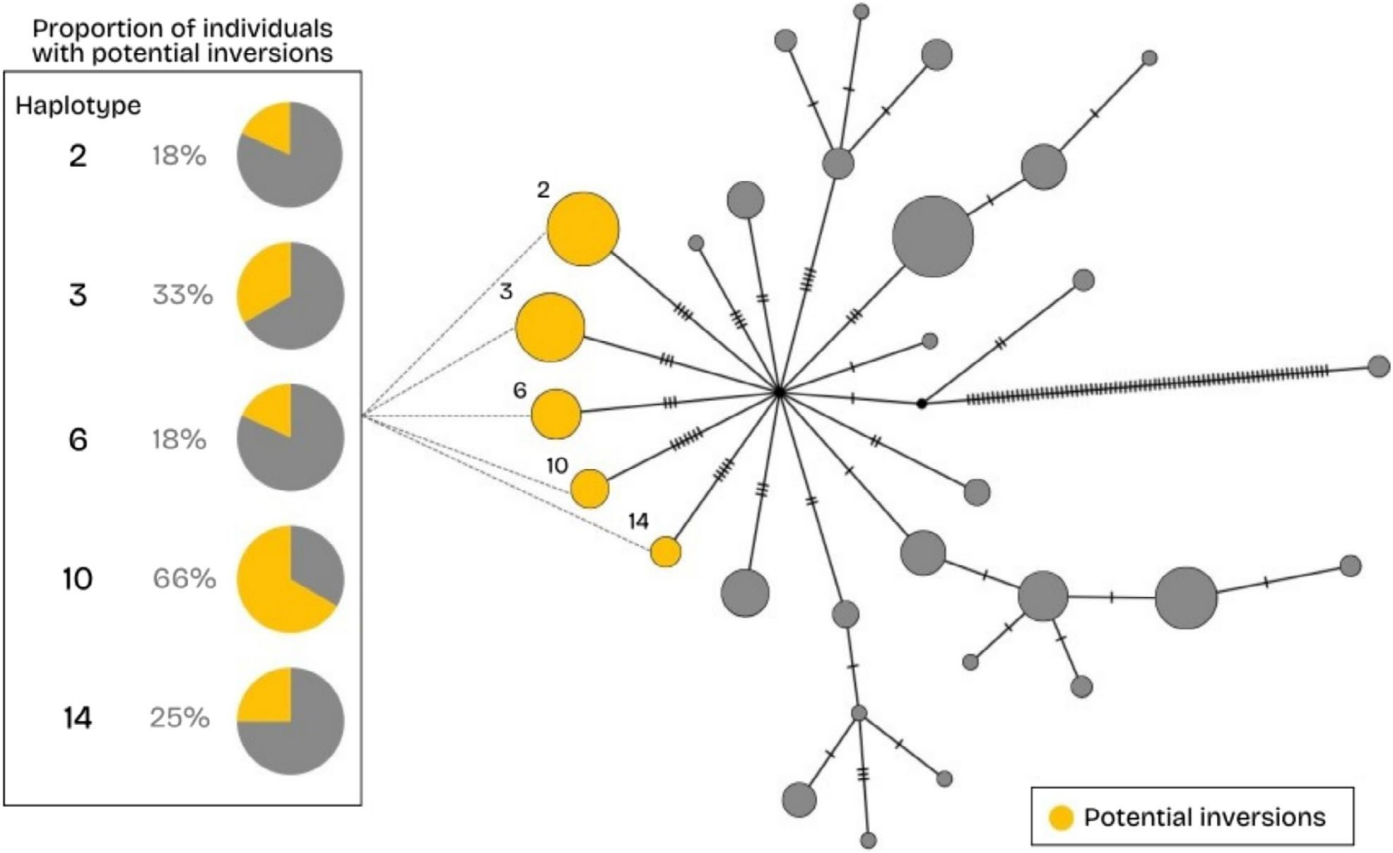
Median‐joining haplotype network associating the 30 mitochondrial DNA haplotypes identified among North Mountain Brook Trout. Mutational steps are represented by hashmarks on the edges and nodes scaled to represent frequency. Haplotypes containing individuals with potential inversions are highlighted in yellow and the frequency of these individuals within each haplotype is shown. Five haplotypes are present in individuals with and without potential inversions.

Thirty mitochondrial DNA haplotypes were identified in this system, and the median‐joining haplotype network showed 29 of these 30 radiating from a single node by 1–7 mutational steps (Figure 6), suggesting they share recent common ancestry or lineage. The same pattern was observed in the neighbor‐joining dendrogram (Figure S8). Individuals with potential inversions were identified in haplotypes 2, 3, 6, 10, and 14 (Figure 6). These haplotypes occur in both individuals carrying the potential inversions as well as in those lacking them (Figure 6). These results suggest the potential inversions are recently derived and not the result of long‐term population structure, such as refugial isolation. Furthermore, these haplotypes do not appear to share a common ancestor more recent than the potential ancestor of all haplotypes in the network (Figure 6) and do not form a distinct clade in the neighbor‐joining tree (Figure S8). Haplotypes 6 and 14 separately share more recent common ancestry with haplotypes not containing potential inversions than with other haplotypes containing potential inversions (Figure S8).

### 3.5 Gene Annotation

Two hundred and thirteen genes and biological processes/pathways were identified among the four potential inversion regions (Tables S3 and S4). The inversion in chromosome 31 is the largest, containing 110 different genes and 13 GO terms (Tables S3 and S4). In comparison, the smallest inversion in chromosome 19 contained only 12 genes, and no GO terms were identified (Tables S3 and S4). Chromosomes 12 and 27 are of similar size, and four GO terms were identified in each, while chromosome 12 contains 66 genes and chromosome 27 contains 25 genes (Tables S3 and S4).

### 3.6 Water Temperature

Among the five of nine streams for temperature was recorded, Healeys and Poole brook (westernmost streams) consistently had higher maximum daily water temperatures, with Healeys reaching the highest, while Ross Creek (easternmost) consistently had the lowest temperatures (Figure S6).

### 3.7 Environmental Association

**FIGURE 7:**
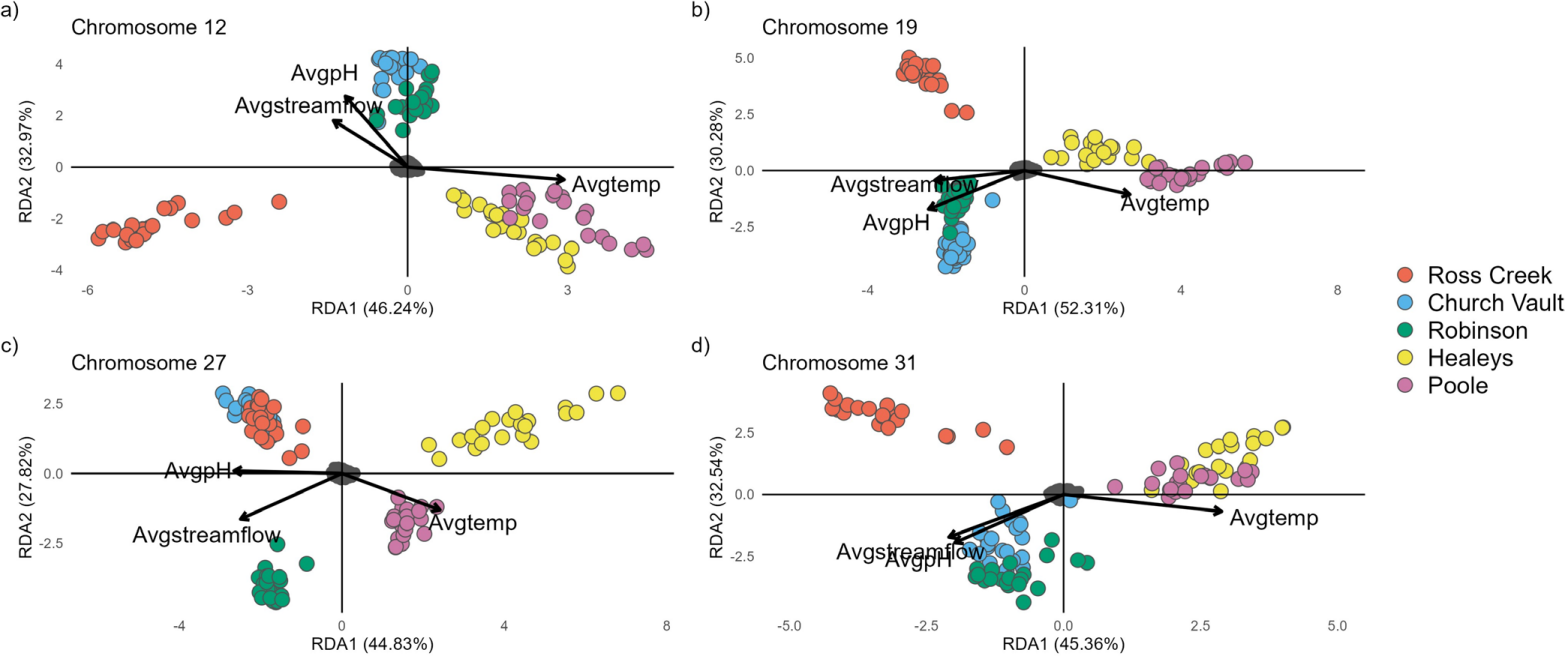
(a–d) RDAs across potential inversion chromosomes where the constrained variables in the model are average water temperature, average pH, and average streamflow. Streams are ordered from east to west along the North Mountain, Nova Scotia.

All redundancy analyses (RDAs) show a positive association with water temperature as indicated by the direction and angle of vectors to individuals from Healeys and Poole (Figure 7a–d). pH and streamflow show positive associations with individuals from Church Vault and Robinson for three of the four potential chromosomal inversions (Figure 7a–d). Regardless of the direction and angle of the associations, the environmental variables explained a low percentage of the variation that did not exceed 27.2% from adjusted *R* ^2^ (Table 2) and all axes of RDAs are significant (*p* < 0.001). Outlier SNPs from RDAs were identified on all four chromosomes (Table 3). In chromosome 31, most outlier SNPs are within the potential inversion region (Table 3).

**TABLE 2:** Adjusted *r* ^2^ values produced for each RDA of potential inversion chromosomes. RDA models were done using average streamflow, average pH and average temperature.

Chromosome	Adjusted *R* ^2^
12	0.272
19	0.265
27	0.270
31	0.254

**TABLE 3:** Number of significant outlier SNPs identified from RDAs as three standard deviations from the mean.

Chromosome	Number of outlier SNPs	Number of outlier SNPs within potential inversions
12	129	18
19	94	30
27	103	50
31	95	74

## 4 Discussion

Our initial goal was to assess population genomic structure including evidence of neutral and functional differentiation among nine neighboring but isolated Brook Trout small populations. This effort led to the discovery of structural variants in a subset of these populations. Four large haploblocks identified as potential chromosomal inversions representing 2.72% of the Brook Trout genome were discovered among three of the nine Brook Trout populations examined. All Brook Trout originate from one lineage, and redundancy analysis provides evidence for possible local adaptation. Linkage disequilibrium patterns are similar among chromosomes 12, 19, 27, and 31, showing individuals from western streams exhibiting large linkage disequilibrium blocks while those in the east do not (Figure 3). PCAs of potential inversion regions show the three groups suggestive of heterokaryotypes and homokaryotypes with and without potential inversions (Figures 4 and 5). The low frequency of individuals with potential inversions from only the western populations suggests that the potential inversions are rare in the study system of the North Mountain, Nova Scotia. The population structure of these Brook Trout is not strictly shaped by the presence of potential inversions as there are genome‐wide high levels of *F*_ST_ and a PC plot excluding SNPs in linkage shows a very similar pattern to that with all 2.6 million SNPs (Figure 2; Figures S2 and S3).

### 4.1 Did the Inversions Originate Post‐Colonization?

The presence of potential inversions only in the western populations suggests that local deglaciation of the North Mountain, Nova Scotia, and colonization history might have influenced this pattern. Different patterns of ice advance and retreat took place during the late Wisconsinan phase approximately 14,000–10,000 years before present (Stea et al. 1998). During this period, different marine incursion and recession events generated a post‐glacial history that led the western streams (Healeys, Sheep Shearer, Poole) to deglaciate first (Shaw et al. 2006; Stea 2004; John Gosse, personal communications, November 2022). Glaciation and postglacial colonization history play a role in population distribution and structure among fishes (Bernatchez and Wilson 1998; Ruzzante et al. 2020) as has also been seen in Brook Trout (Ferchaud et al. 2020; Pilgrim et al. 2012). In addition, eastern Canada exhibits evidence of having been colonized by Brook Trout originating from Atlantic and Acadian refugia (Danzmann et al. 1998) but mitochondrial DNA evidence suggests that Brook Trout from New Brunswick and Cape Breton, Nova Scotia, originate from a single lineage (Jones et al. 1996). To our knowledge, this is the first time Brook Trout from the North Mountain are included in mitochondrial DNA analyses, and the evidence we report in the present study suggests they originate from a single lineage as well (Figure 6; Figure S8). The group of individuals carrying the potential inversions exhibits five different mitochondrial DNA haplotypes, but these haplotypes are shared with individuals that do not exhibit the potential inversions, suggesting the potential inversions took place post‐colonization. Both the mitochondrial haplotype network and phylogeny suggest these populations descend from the same ancestor, likely from a single glacial refugium (Figure 6; Figure S8). If the potential inversions were present before recolonization, all lineages would have possessed the potential inversions at some time in the past, and most (excepting 2, 3, 6, 10, and 14, which have the potential inversions contemporarily) would have lost them. There would have had to have been several loss events at different time points to account for the phylogeny, given that some haplotypes with potential inversions are more derived than others and are sister to potentially non‐inverted haplotypes (see haplotypes 6 and 14; Figure S8). The most parsimonious explanation given the data presented here is that the potential inversions arose after the westernmost populations were established. We assumed individuals with inversions are those with the lower observed heterozygosity between the two homokaryotype groups (Figure 5). Under this assumption, the potential inversions would be the derived state as we expect a younger arrangement to have relatively low diversity due to a shorter time for accumulation of mutations (Faria, Johannesson, et al. 2019).

### 4.2 Local Adaptation Among Potential Inversions

Potential inversions among the three westernmost populations could contribute to local adaptation as shown by the redundancy analysis (Figure 7). Redundancy analysis across potential inversion chromosomes on a subset of populations for which we had environmental data shows average water temperature corresponding with genetic variation in Brook Trout from Healeys and Poole (Figure 7).

Two hundred and thirteen genes were identified within the four potential inversions, and gene annotation suggests some key pathways of significance, such as response to hypoxia, response to oxidative stress, and other metabolic‐specific processes (Tables S3 and S4). A total of 172 statistically significant SNPs were found within potential inversion regions based on redundancy analysis, and some are found within gene regions on chromosomes 19, 27, and 31 (Table 3). One such SNP was found located within the pou1f1 gene on chromosome 19, which controls expression of growth and other hormones in teleost fishes (Wang et al. 2017) and was most associated with average water temperature. SNPs were also found within the ttn.1 gene and were most associated with temperature and streamflow. The ttn.1 gene encodes the titin protein, which is involved in muscle development and has been linked to muscle‐specific genetic diseases among zebrafish ( *Danio rerio* ) (Santiago et al. 2021; Steffen et al. 2007). Of particular interest was the finding of significant SNPs within the mapk13 gene on chromosome 31. The mapk gene family plays a large role in cell growth and stress responses to the environment and was found to be expressed at high levels in the gill tissue of black rockfish ( *Sebastes melanops* ), suggestive of a response to osmotic stress (Zhang et al. 2023). A similar finding was discovered among northern snakehead ( *Channa argus* ) (Sun et al. 2025). The SNPs located within the mapk13 gene were most associated with average streamflow, and lower oxygen in streams is expected to correlate with lower streamflow. Maximum daily water temperatures in Healeys and Poole, where potential inversions were found in our study, were the highest among the five populations for which we had temperature data, sometimes exceeding 20°C (Figure S1) which approaches Brook Trout's upper limit for growth (Chadwick and McCormick 2017) and preferred temperature (Smith and Ridgway 2019). This could generate stress responses, of which the roles of genes such as mapk13 would provide an adaptive advantage. Common garden experiments need to be performed to understand the response to the environment, but redundancy analysis does provide support for local adaptation of potential inversions discovered here.

The findings of four structural variants which could be potential inversions in only western streams are critical to our understanding of intraspecific population variation and conservation of such populations. Recent research on Brook Trout in Nova Scotia showed that there is limited introgression between stocked and wild populations and that there is strong population structuring (Lehnert et al. 2020). Highly structured populations are very common among Brook Trout in other areas of its native range (Castric et al. 2001; Erdman et al. 2022; Morgan II et al. 2021). Brook Trout have historically been stocked in Nova Scotia to sustain fisheries and restore populations (Nova Scotia Department of Agriculture and Fisheries Inland Fisheries Division 2005). Research by Lehnert et al. (2020) shows limited introgression with some in the Margaree River system in which a locally derived strain is used, suggesting these individuals likely have an adaptive advantage. Other research also finds limited hatchery introgression with wild populations (Erdman et al. 2022; White et al. 2018) but recent research shows introgression can be affected by habitat (Bruce et al. 2020). If populations of Brook Trout in Nova Scotia are locally adapted as evidence here suggests is likely, introgression from stocking could become problematic for intraspecific population variation. Locally adapted genetic diversity could be reduced by admixture (Ryman and Laikre 1991) from introgression for the study populations due to their small effective population sizes (Ruzzante et al. 2016, 2025). This would be of particular importance if these potential inversions existed among other populations of Brook Trout in the province, and the development of a genetic monitoring program could bolster our understanding of such effects. This study also has implications at a broader scale in terms of the conservation of small populations from the perspective of conservation units and designatable units representing intraspecific population variation. While Brook Trout are not listed under the Canadian federal Species at Risk Act, our study provides additional reasoning for the incorporation of intraspecific genomic‐based adaptation such as structural variants in delineating designatable units which could promote the maintenance of genetically diverse populations. Other research on salmonids has already begun to assess how, for example, large effect loci could or could not be used to designate conservation units among Chinook Salmon ( *Oncorhynchus tshawytscha* ) and Steelhead ( *Oncorhynchus mykiss* ) (Waples et al. 2022). In addition, recent research has used evidence of genomic‐based adaptation such as structural variants to define designatable units in eastern Canada for anadromous Atlantic Salmon ( *Salmo salar* ) (Lehnert et al. 2023). The potential inversions in the present study were discovered only in the westernmost populations which differ in a number of environmental variables from the rest of the populations examined. These results, along with the functions of genes encountered within the potential inversions, lend support to the suggestion of an adaptive role for the structural variants, which should thus be considered in the delineation of conservation units for Brook Trout.

The use of whole genome sequencing has facilitated the detection of structural variants including chromosomal inversions (Liu et al. 2021; Waldbieser et al. 2023) and indirect detection methods including the use of high linkage disequilibrium regions can be biased towards the preferential consideration of large genomic regions. Large structural variants typically hold more SNPs than small regions and thus could be adaptive and have more impact on frequency, establishment, and persistence (Hale et al. 2021; Wellenreuther and Bernatchez 2018). While the size of potential inversions can affect their evolution (Connallon and Olito 2022), populations with small effective size will likely be exposed to high levels of genetic drift leading to potentially false inference of adaptive loci (Leigh et al. 2021). High levels of linkage disequilibrium can also be evidence of other types of structural variants such as copy number variants and fusions, and in addition could be the result of other factors such as selective sweeps (Mérot et al. 2020). In the present study, however, further support for inversions arises from the relatively low observed heterozygosity exhibited by both homokaryotypes. It is important to acknowledge that this pattern cannot rule out fusions and haploblocks as low observed heterozygosity can also be evidence for these (Lamichhaney and Andersson 2019; Wellband et al. 2019). Long read sequencing would need to be performed, however, to confirm these are indeed chromosomal inversions (Mérot et al. 2020; Warburton and Sebra 2023). In addition, gene expression and common garden experiments (Bernatchez et al. 2024; Hämälä et al. 2021; Ma et al. 2024; Thomas et al. 2022) can help identify the role of genes within potential inversions. Conversely, interpretation may be complicated by phenotypic plasticity influencing ecological fitness (Wood et al. 2014, 2016; Wood and Fraser 2015).

In conclusion, this study provides evidence of four potential inversions that are unique to western populations of Brook Trout in the North Mountain region of Nova Scotia, and which do not shape genome‐wide patterns of differentiation. Understanding the role of these potential inversions and interpopulation variation for local adaptation and fitness could provide an important avenue of research for the conservation of Brook Trout. More effort could be placed on understanding how structural variants and inversions could be advantageous to Brook Trout, a thermally sensitive species that faces challenges due to ongoing temperature increases across the species range.

## Author Contributions

**Cait M. Nemeczek:** data curation (lead), formal analysis (lead), investigation (lead), methodology (lead), project administration (lead), writing – original draft (lead), writing – review and editing (lead). **M. Lisette Delgado:** supervision (equal), writing – review and editing (equal). **Meg E. Smith:** data curation (equal), formal analysis (equal), investigation (equal), visualization (supporting), writing – original draft (equal), writing – review and editing (supporting). **John MacMillan:** conceptualization (equal), methodology (equal). **Mallory Van Wyngaarden:** resources (equal), supervision (equal). **Daniel E. Ruzzante:** conceptualization (equal), funding acquisition (lead), methodology (equal), project administration (equal), resources (lead), supervision (lead), writing – review and editing (equal).

## Disclosure

*Benefit‐Sharing Statement*: Research collaboration was developed with co‐authors from Nova Scotia Department of Fisheries and Aquaculture who have connections with the sport fishing associations. This research addresses a priority concern, in this case the conservation of Brook Trout, a top sportfish with a narrow thermal tolerance. The contributions of all individuals to this research are appropriately described in Acknowledgments and Author Contributions.

## Conflicts of Interest

The authors declare no conflicts of interest.

## Supporting information


**Table S1:** Physical characteristics of the nine streams sampled for Brook Trout in the North Mountain, Annapolis Valley, Nova Scotia and average and median fork lengths of 40 Brook Trout sampled from each stream.
**Table S2:** Average pH calculated from pH measured in the middle and at both banks of each stream.
**Table S3:** Biological processes and pathways involved in statistically significant gene symbols from chromosomes 12, 19, 27, and 31.
**Table S4:** Genes identified within potential inversion regions on each chromosome using the UCSC genome browser.
**Figure S1:** Maximum daily water temperatures.
**Figure S2:** Manhattan plot of *F*_ST_ between individuals from populations with potential inversions (Healeys, Poole, Sheep Shearer) and individuals from populations without potential inversions (Ross Creek, Woodsworth, Blackhole, Church Vault, Robinson, Saunders) in 10,000 bp windows with a 10,000 bp slide. *F*_ST_ was calculated from the folded site frequency spectrum of sample allele frequencies for individuals from the three populations with potential inversions and the six without potential inversions.
**Figure S3:** PCA with genotype likelihoods for *N* = 192 Brook Trout produced from the covariance matrix of 352,578 SNPs representing those after linkage disequilibrium pruning and removing potential inversion chromosomes.
**Figure S4:** Observed proportion of heterozygosity across the genome (2.6 million SNPs) for 21 individuals from Poole, Healeys, and Sheep Shearer that have potential inversions on chromosomes 12, 19, 27, or 31. These individuals are the same as from Figure 5 of homokaryotypes with potential inversions.
**Figure S5:** Average sequencing depth per stream (population) calculated at all positions covered by reads using the Brook Trout ( *Salvelinus fontinalis* ) reference genome.
**Figure S6:** Heatmaps of *r* ^2^ from linkage disequilibrium calculations for 192 Brook Trout on all 42 chromosomes in the Brook Trout genome. The second percentile of *r* ^2^ values in 250 kb windows were used for plotting. High *r* ^2^ values and dark colors represent areas on a chromosome with high linkage disequilibrium.
**Figure S7:** Average observed proportion of heterozygotes (*H*_obs_) across entire chromosomes of individuals from the three different potential inversion groups from PCAs of linkage disequilibrium blocks. All individuals from each of the potential inverted and non‐inverted homokaryotypes and heterokaryotypes were used to calculate *H*_obs_. Average *H*_obs_ was calculated in 10 kb windows using a 10 kb slide and points on the plot represent H_obs_ among groups for SNPs in these windows.
**Figure S8:** (A) Neighbor‐joining tree containing 30 haplotype sequences identified among North Mountain Brook Trout ( *Salvelinus fontinalis* ) populations, the Brook Trout reference mitogenome and the Lake Trout ( *Salvelinus namaycush* ) reference mitogenome as the outgroup. Bootstraps indicated at branch nodes. (B) Branch lengths scaled to represent sequence divergence.

## Data Availability

Individual bam files used for potential inversion analysis and phylogenetic analysis are available on the NCBI SRA database under the BioProject accession number PRJNA1144401. All scripts used are available on https://github.com/cnemeczek/ANGSD‐BrookTrout.
