# Supplementary material for: The Potential Role of Haploblocks in the Persistence of Small, Isolated Populations of Brook Trout (Salvelinus fontinalis)
Source: Ecol Evol. 2025 Sep 15;15(9):e72075. doi: 10.1002/ece3.72075 (PMC12434321; doi:10.1002/ece3.72075)
Supplement: Supplementary file 1 — Table S1: Physical characteristics of the nine streams sampled for Brook Trout in the North Mountain, Annapolis Valley, Nova Scotia and average and median fork lengths of 40 Brook Trout sampled from each stream. Table S2: Average pH calculated from pH measured in the middle and at both banks of each stream. Table S3: Biological processes and pathways involved in statistically significant gene symbols from chromosomes 12, 19, 27, and 31. Table S4: Genes identified within potential inversion regions on each chromosome using the UCSC genome browser. Figure S1: Maximum daily water temperatures. Figure S2: Manhattan plot of F ST between individuals from populations with potential inversions (Healeys, Poole, Sheep Shearer) and individuals from populations without potential inversions (Ross Creek, Woodsworth, Blackhole, Church Vault, Robinson, Saunders) in 10,000 bp windows with a 10,000 bp slide. F ST was calculated from the folded site frequency spectrum of sample allele frequencies for individuals from the three populations with potential inversions and the six without potential inversions. Figure S3: PCA with genotype likelihoods for N = 192 Brook Trout produced from the covariance matrix of 352,578 SNPs representing those after linkage disequilibrium pruning and removing potential inversion chromosomes. Figure S4: Observed proportion of heterozygosity across the genome (2.6 million SNPs) for 21 individuals from Poole, Healeys, and Sheep Shearer that have potential inversions on chromosomes 12, 19, 27, or 31. These individuals are the same as from Figure 5 of homokaryotypes with potential inversions. Figure S5: Average sequencing depth per stream (population) calculated at all positions covered by reads using the Brook Trout ( Salvelinus fontinalis ) reference genome. Figure S6: Heatmaps of r 2 from linkage disequilibrium calculations for 192 Brook Trout on all 42 chromosomes in the Brook Trout genome. The second percentile of r 2 values in 250 kb windows were used for [file ECE3-15-e72075-s001.docx]

The potential role of haploblocks in the persistence of small, isolated populations of Brook Trout (*Salvelinus fontinalis*)

Cait M. Nemeczek^1*^, M. Lisette Delgado^1^, Meg E. Smith^1^, John MacMillan^2^, Mallory Van Wyngaarden^1+^, Daniel E. Ruzzante^1*^

# Supplemental

Table S1 Physical characteristics of the nine streams sampled for Brook Trout in the North Mountain, Annapolis Valley, Nova Scotia and average and median fork lengths of 40 Brook Trout sampled from each stream.

| Stream | Surficial Geology | Average fork length (cm) | Median fork length (cm) |
| --- | --- | --- | --- |
| Ross Creek | Scoured layer of glacial till | 10.33 | 9.5 |
| Woodworth | Scoured layer of glacial till | 11.01 | 10.8 |
| Black Hole | Scoured layer of glacial till | 11.77 | 10.6 |
| Church Vault | Thick stony-granite derived till | 11.32 | 11 |
| Saunders | Thick stony-granite derived till | 11.63 | 11 |
| Robinson | Thick stony-granite derived till | 10.80 | 10.45 |
| Sheep Shearer | Silt | 15.11 | 14.4 |
| Healeys | Silt | 11.48 | 10.95 |
| Poole | Silt | 12.87 | 12.4 |

Table S2 Average pH calculated from pH measured in the middle and at both banks of each stream

where streamflow was measured using a pHep^+^ H198108 (Hanna instruments) once a month.

| Stream | Average pH July 14 2021 | Average pH August 9 2021 | Average pH September 13 2021 | Average pH October 6 2021 | Average pH November 17 2021 |
| --- | --- | --- | --- | --- | --- |
| Ross Creek | 7.60 | 7.79 | 7.73 | 7.67 | 7.62 |
| Woodworth Creek | 7.78 | 7.87 | 7.84 | 7.65 | 7.56 |
| Black Hole Brook | 7.74 | 7.83 | 7.92 | 7.80 | 7.64 |
| Church Vault Brook | 8.00 | 8.10 | 7.98 | 8.01 | 7.70 |
| Saunders Brook | 8.09 | 8.16 | 8.00 | 8.08 | 7.82 |
| Robinson Brook | 7.88 | 7.81 | 7.73 | 7.86 | 7.86 |
| Sheep Shearer Brook | 7.84 | 7.51 | 7.50 | 7.44 | 7.70 |
| Healeys Brook | 7.42 | 7.55 | 7.47 | 7.36 | 7.77 |
| Poole | 7.59 | 7.50 | 7.43 | 7.17 | 7.77 |

Table S3 Biological processes and pathways involved in statistically significant gene symbols from chromosomes 12, 19, 27, and 31.

Processes with a -log10(P) greater than two were included in the table and greater than three for chromosome 31.

| Chromosome | GO biological process, KEGG Pathways, Reactome Gene Sets |
| --- | --- |
| 12 | Cytokinetic process, cilium assembly, carbohydrate metabolic processes |
| 19 | none |
| 27 | Mitochondrion organization, negative regulation of intracellular signal transduction |
| 31 | Negative regulation of cellular catabolic process, regulation of TP53 activity, positive regulation of autophagy of mitochondrion in response to mitochondrion depolarization, PID ERBB2 ERBB3 pathway, 1p36 copy number variation syndrome, ERAD pathway, response to hypoxia, glutathione metabolism, positive regulation of protein localization, response to oxidative stress, positive regulation of cellular component biogenesis, negative regulation of DNA metabolic process, response to UV |

Table S4 Genes identified within potential inversion regions on each chromosome using the UCSC genome browser.

| Chromosome 12 | Chromosome 19 | Chromosome 27 | Chromosome 31 |
| --- | --- | --- | --- |
| agbl2  arl2bp  armc10  bend7  bhlhe41  btbd11b  c12h15orf40  calub  cax2  ccdc113  cdk10  cfdp1  ch25hl1.1  chd2  chmp1a  chst6  cmip  coq9  exoc3l1  fam185a  gabarapl2  gdpgp1  hydin  kiaa0513  lrrn2  mapk12a  mlc1  neto2b  pdhx  pdp2  pgpep1l  phrf1  plcg2  plin1  pllp  polr2c  prmt7  prr5a  rabl2  rad52  rcn2  rgma  rxylt1  sephs1  si:ch1073-390k14.1  si:ch211-59o9.10  si:dkey-30c15.13  si:dkey-30c15.2  slco3a1a  sntb2  spata2l  spire2  st8sia2  tat  terb1  tmem231  tmem263  tmem60  trabd  tsnaxip1  vps4a  zfpm1  zgc:112052  zgc:56622  zgc:77752  znf276 | arsh  cerkl  dnajc10  hspbap1  itprid2  nifk  pdia5  pou1f1  slc49a4  tfcp2l1  tsn  ttn.1 | acat2  afg1lb  ak9  cep57l1  epm2a  fam166c  fig4a  foxo3b  glrx5  opn8a  opn8b  otofa  ppil6  sash1a  sesn1  slc25a47a  snx3  sod2  tcp1  tdrd6  ubxn2a  utrn  wasf1  wtap  zgc:112001 | acot7  adnpb  agmat  alas2  ano11  apex2  arhgef16  brpf3b  casz1  ccdc30  chchd6a  chrna4b  clstn1  cntn3a.1  cntn3a.2  ctnnbip1  ddx19a  dedd  dffa  dnajc16  dpm1  edem2  efhd2  ela2  ela2l  espn  exosc10  fam217ba  fam43b  fance  fhad1  gata5  gdap1l1  gss  her3  hm13  hspg2  igf3  ints11  kif1b  lamb2  lamb2l  lemd1  lzic  mapk13  masp2  mesd  mfn2  mtor  mul1b  nfascb  nkain5  nmnat1  noc2l  nppal  nppb  otud3  pdyn  pex14  pgd  pik3cd  pink1  plod1a  ppih  ppp1r3da  prdm16  prdm2a  prex1  ptpn22  rer1  rtel1  samd11  si:ch211-167b20.8  si:ch211-218o21.4  si:ch73-206d17.1  si:dkey-178k16.1  si:dkey-32e6.3  si:dkeyp-100a1.6  si:dkeyp-110g5.4  si:rp71-17i16.5  si:rp71-17i16.6  sike1  slc35c2  smim1  srm  taf10  taf11  taf13  tafa5l  tardbpa  tlnrd1  tmco4  tmem201  tmem51a  tnnc2.2  tp73  tpk2  tprg1l  troap  ttll9  ube4b  usp19  vwa1  vwa5b1  wdr13  wrap73  ybx1  zgc:154075  zgc:56699  zmynd12 |


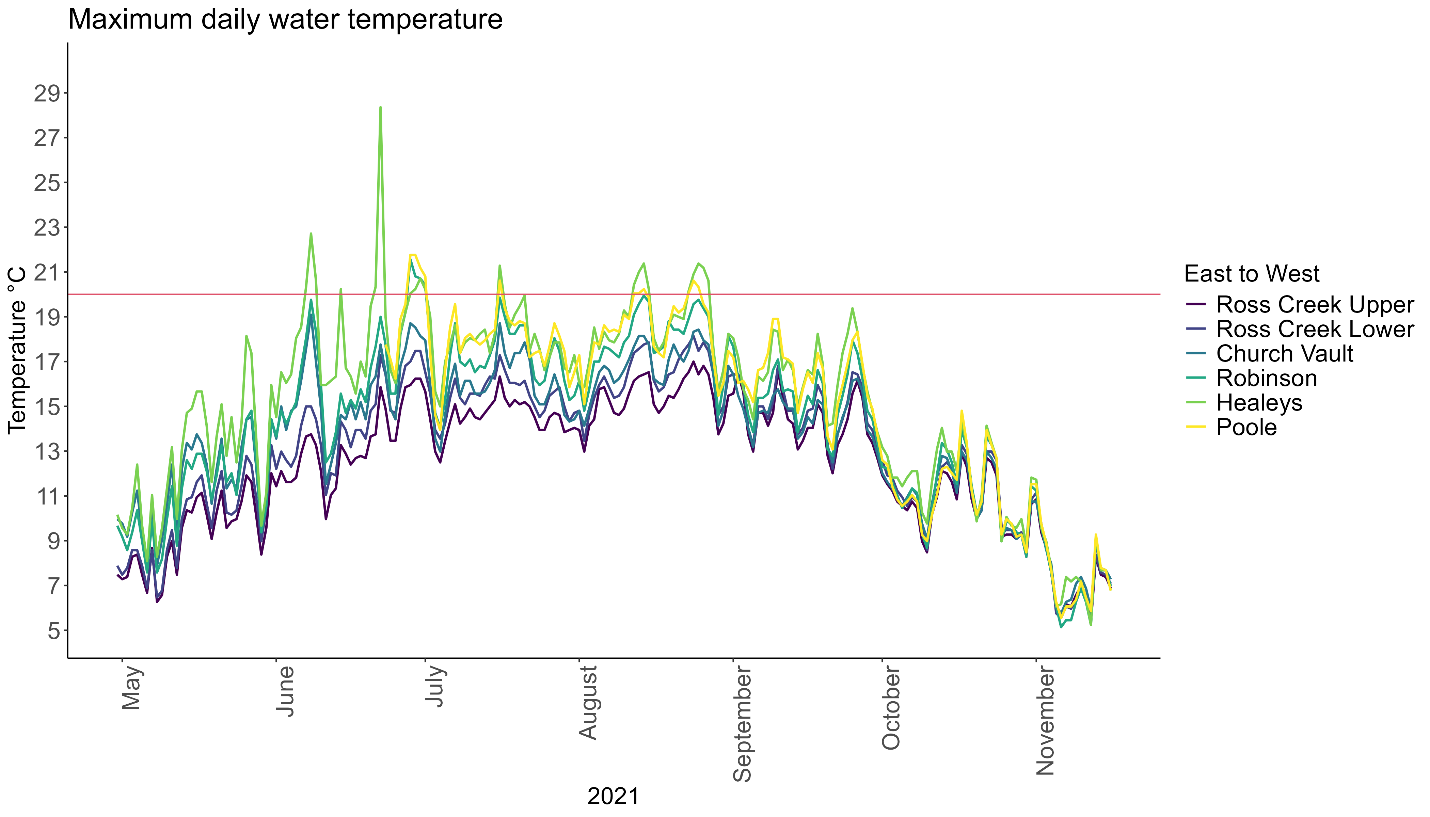


Figure S1 Maximum daily water temperatures

calculated from water temperature recorded every two hours from May-November 2021 with HOBO pendant loggers. Temperature was recorded in a subset of the nine streams. The red line represents temperature that Brook Trout avoid.


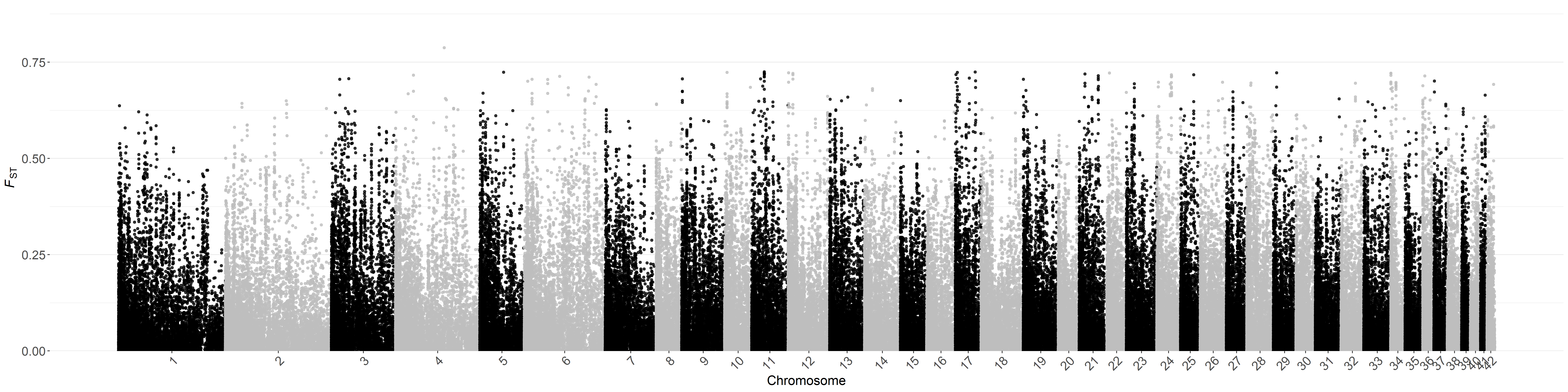


Figure S2 Manhattan plot of F_ST_ between individuals from populations with potential inversions (Healeys, Poole, Sheep Shearer) and individuals from populations without potential inversions (Ross Creek, Woodsworth, Blackhole, Church Vault, Robinson, Saunders) in 10,000bp windows with a 10,000bp slide. F_ST_ was calculated from the folded site frequency spectrum of sample allele frequencies for individuals from the three populations with potential inversions and the six without potential inversions.


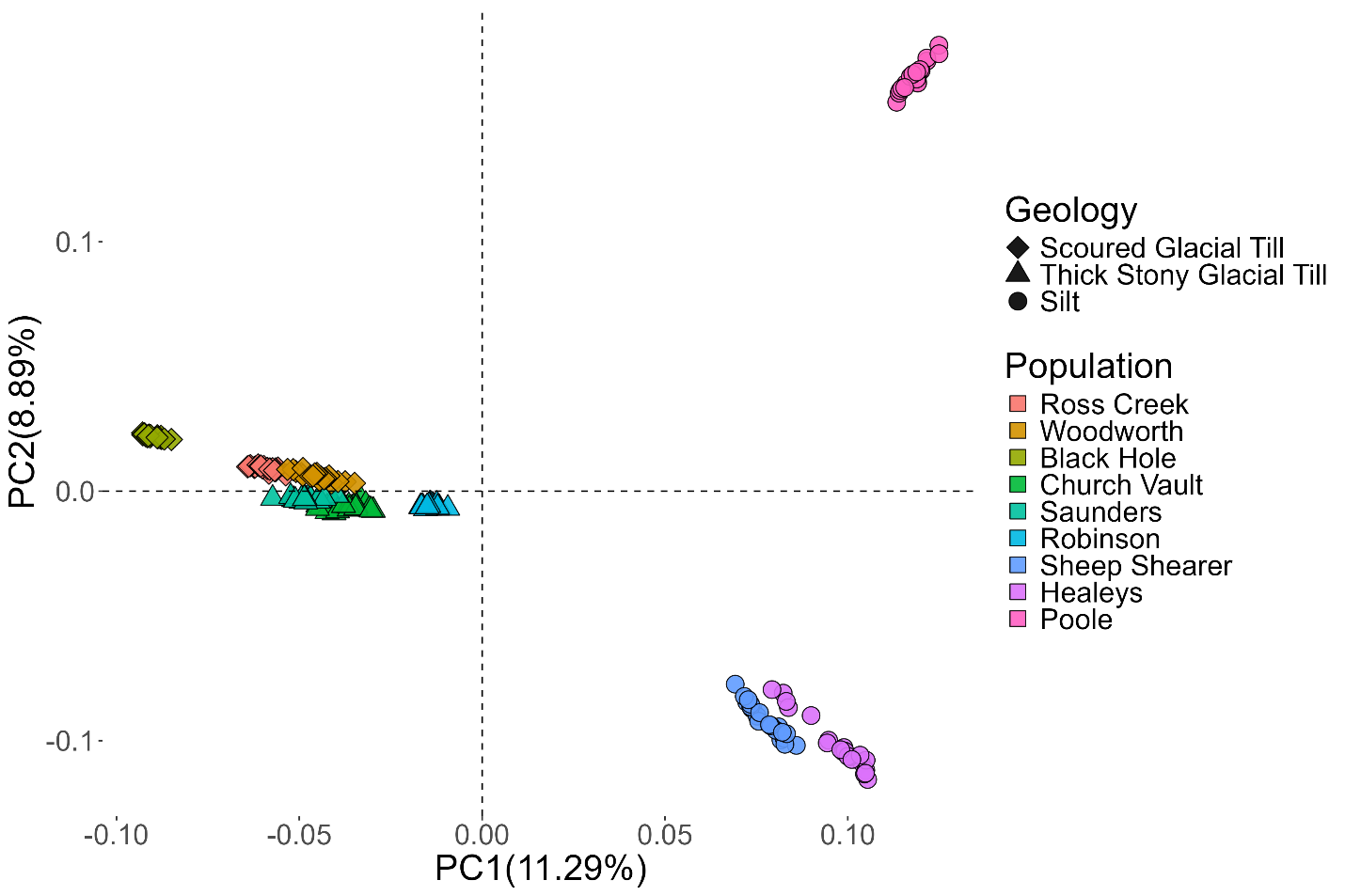


Figure S3 PCA with genotype likelihoods for N=192 Brook Trout produced from the covariance matrix of 352,578 SNPs representing those after linkage disequilibrium pruning and removing potential inversion chromosomes.


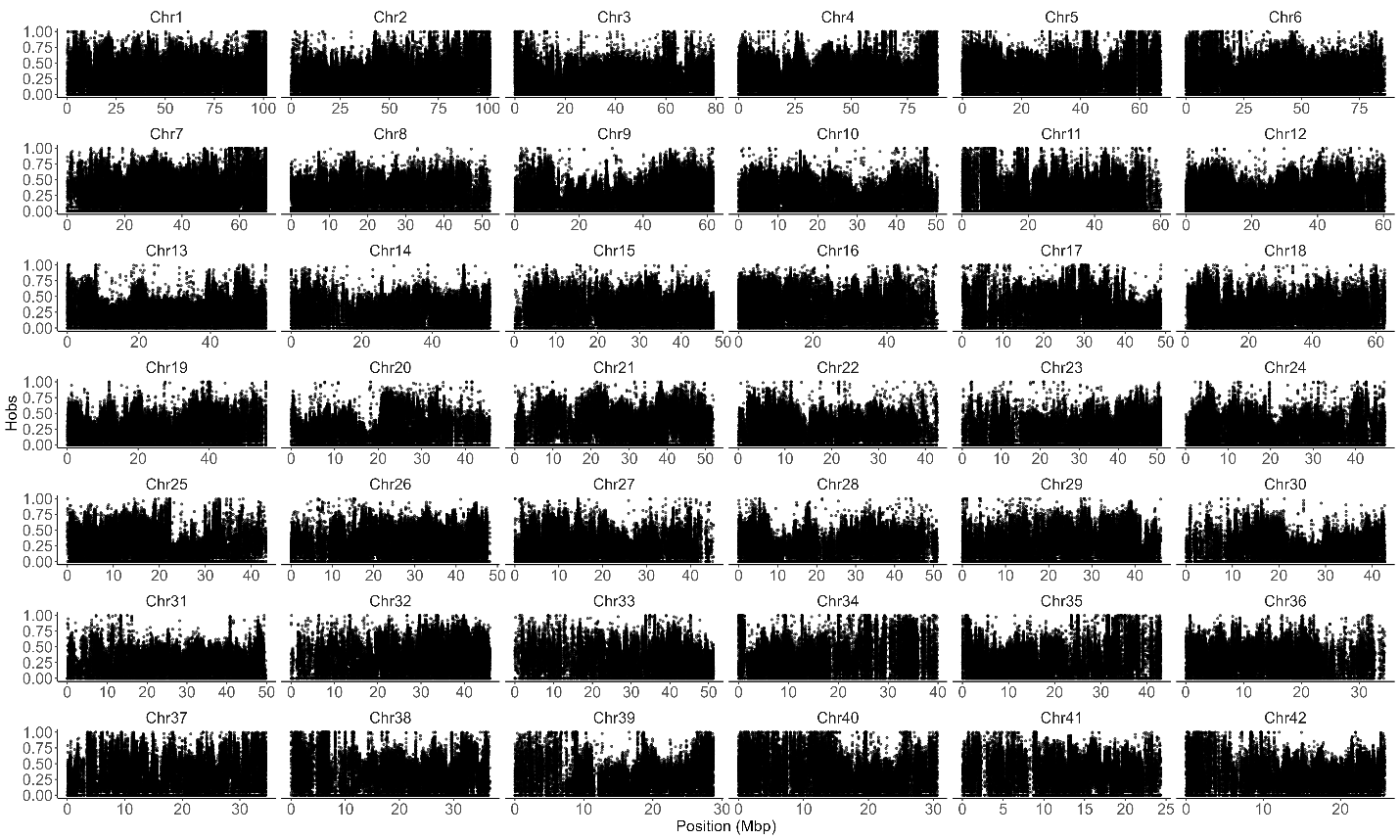


Figure S4 Observed proportion of heterozygosity across the genome (2.6 million SNPs) for 21 individuals from Poole, Healeys, and Sheep Shearer that have potential inversions on chromosomes 12, 19, 27, or 31. These individuals are the same as from Figure 5 of homokaryotypes with potential inversions.


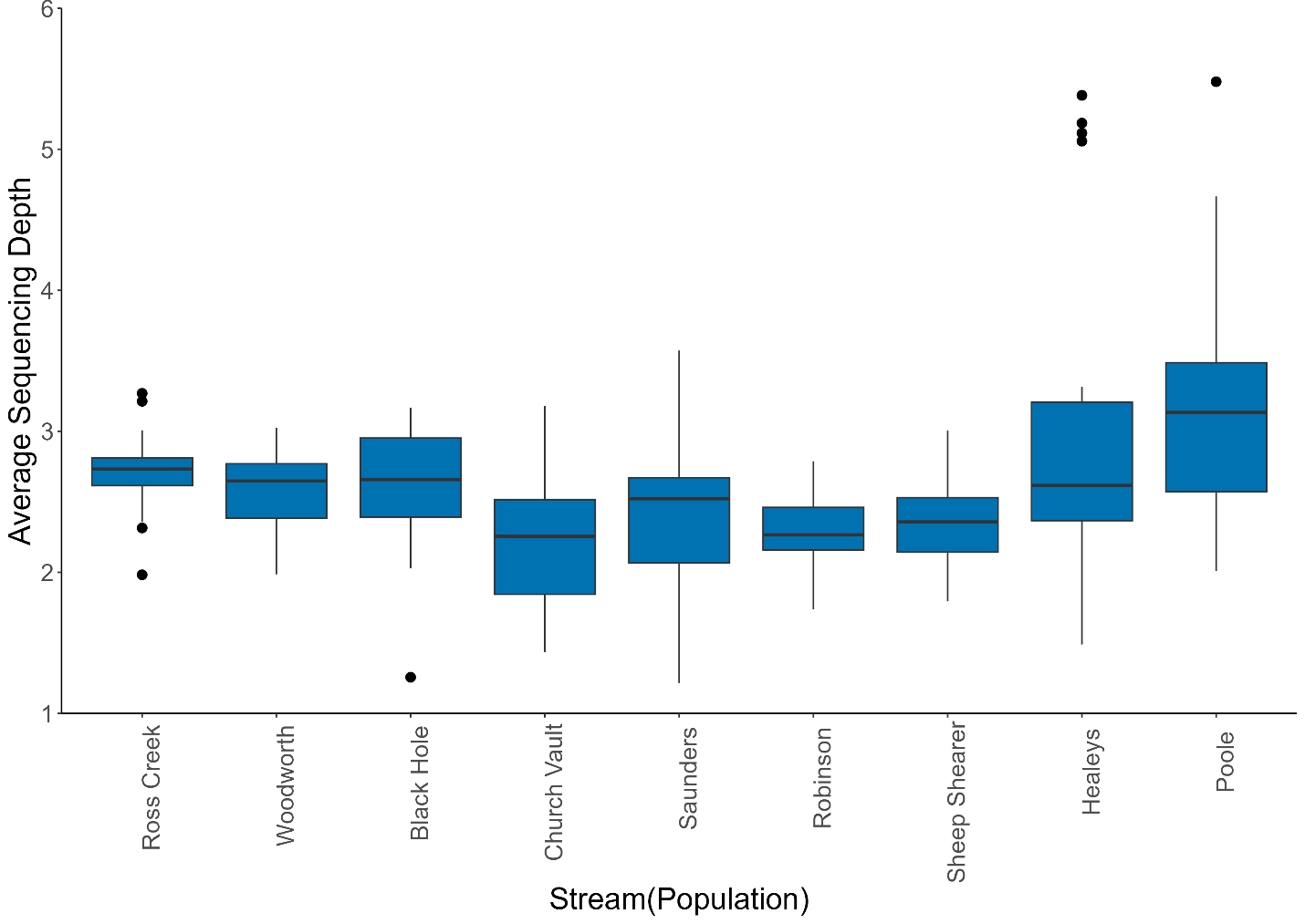


Figure S5 Average sequencing depth per stream (population) calculated at all positions covered by reads using the Brook Trout (Salvelinus fontinalis) reference genome.

Error bars are 95% confidence intervals.


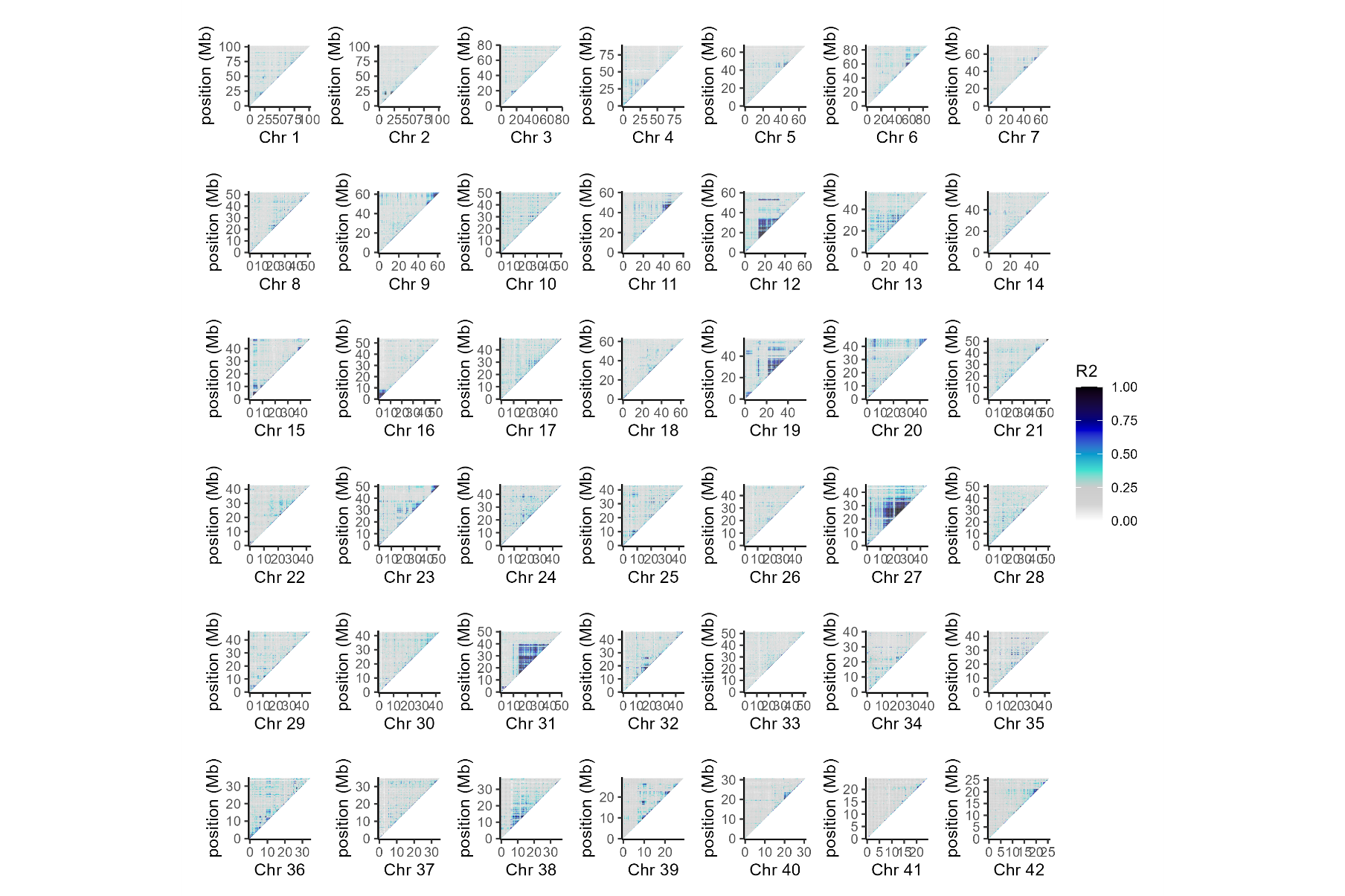


Figure S6 Heatmaps of r^2^ from linkage disequilibrium calculations for 192 Brook Trout on all 42 chromosomes in the Brook Trout genome. The second percentile of r^2^ values in 250kb windows were used for plotting. High r^2^ values and dark colours represent areas on a chromosome with high linkage disequilibrium.


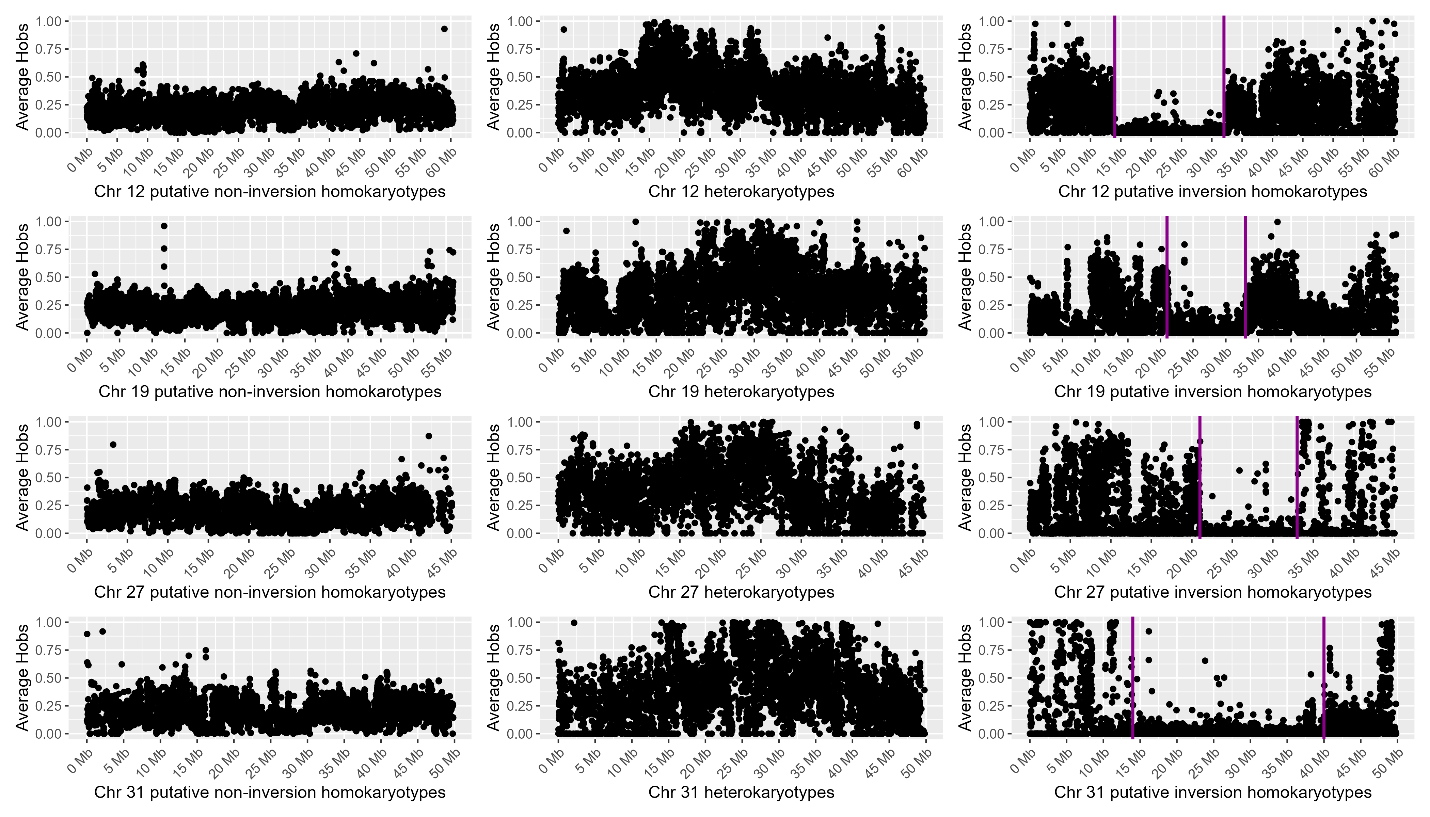


Figure S7 Average observed proportion of heterozygotes (H_obs_) across entire chromosomes of individuals from the 3 different potential inversion groups from PCAs of linkage disequilibrium blocks. All individuals from each of the potential inverted and non-inverted homokaryotypes and heterokaryotypes were used to calculate H_obs_. Average H_obs_ was calculated in 10kb windows using a 10kb slide and points on the plot represent H_obs_ among groups for SNPs in these windows.


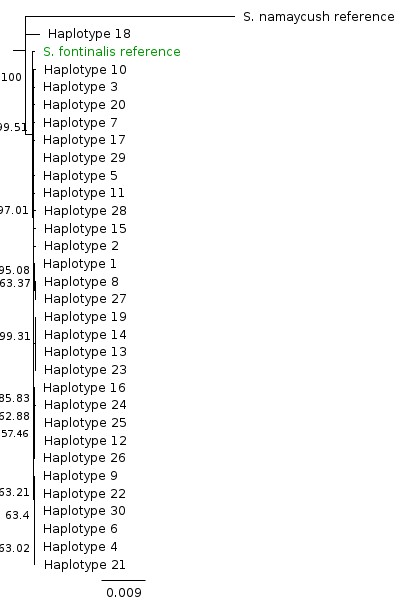


**A**

**B**


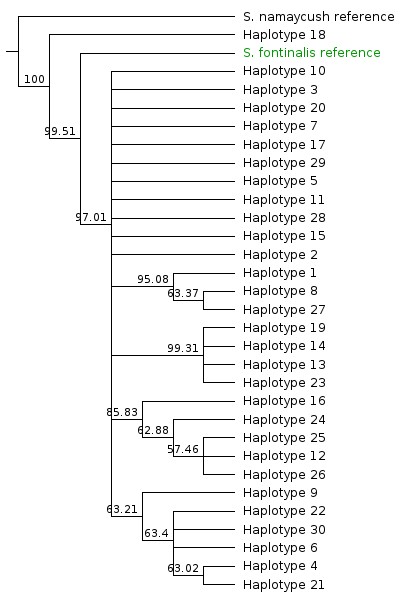


Figure S8 (A) Neighbour-joining tree containing 30 haplotype sequences identified among North Mountain Brook Trout (*Salvelinus fontinalis*) populations, the Brook Trout reference mitogenome and the Lake Trout (*Salvelinus namaycush*) reference mitogenome as the outgroup. Bootstraps indicated at branch nodes. (B) Branch lengths scaled to represent sequence divergence.
